# Exploring and adjusting for potential learning effects in ROLARR: a randomised controlled trial comparing robotic-assisted vs. standard laparoscopic surgery for rectal cancer resection

**DOI:** 10.1186/s13063-018-2726-0

**Published:** 2018-06-27

**Authors:** Neil Corrigan, Helen Marshall, Julie Croft, Joanne Copeland, David Jayne, Julia Brown

**Affiliations:** 1 Clinical Trials Research Unit, Leeds Institute of Clinical Trials Research, University of Leeds, Leeds, LS2 9JT UK; 2 Department of Academic Surgery, Leeds Institute of Biological and Clinical Sciences, Clinical Sciences Building, University of Leeds, St. James’s University Hospital, Leeds, LS9 7TF UK

**Keywords:** Randomised controlled trials, Surgery, Learning curve, Learning effects

## Abstract

**Background:**

Commonly in surgical randomised controlled trials (RCT) the experimental treatment is a relatively new technique which the surgeons may still be learning, while the control is a well-established standard. This can lead to biased comparisons between treatments. In this paper we discuss the implementation of approaches for addressing this issue in the ROLARR trial, and points of consideration for future surgical trials.

**Methods:**

ROLARR was an international, randomised, parallel-group trial comparing robotic vs. laparoscopic surgery for the curative treatment of rectal cancer. The primary endpoint was conversion to open surgery (binary). A surgeon inclusion criterion mandating a minimum level of experience in each technique was incorporated. Additionally, surgeon self-reported data were collected periodically throughout the trial to capture the level of experience of every participating surgeon.

Multi-level logistic regression adjusting for operating surgeon as a random effect is used to estimate the odds ratio for conversion to open surgery between the treatment groups. We present and contrast the results from the primary analysis, which did not account for learning effects, and a sensitivity analysis which did.

**Results:**

The primary analysis yields an estimated odds ratio (robotic/laparoscopic) of 0.614 (95% CI 0.311, 1.211; *p* = 0.16), providing insufficient evidence to conclude superiority of robotic surgery compared to laparoscopic in terms of the risk of conversion to open.

The sensitivity analysis reveals that while participating surgeons in ROLARR were expert at laparoscopic surgery, some, if not all, were still learning robotic surgery. The treatment-effect odds ratio decreases by a factor of 0.341 (95% CI 0.121, 0.960; *p* = 0.042) per unit increase in log-number of previous robotic operations performed by the operating surgeon. The odds ratio for a patient whose operating surgeon has the mean experience level in ROLARR – 152.46 previous laparoscopic, 67.93 previous robotic operations – is 0.40 (95% CI 0.168, 0.953; *p* = 0.039).

**Conclusions:**

In this paper we have demonstrated the implementation of approaches for accounting for learning in a practical example of a surgery RCT analysis. The results demonstrate the value of implementing such approaches, since we have shown that without them the ROLARR analysis would indeed have been confounded by the learning effects.

**Trial registration:**

International Standard Randomised Controlled Trial Number (ISRCTN) registry, ID: ISRCTN80500123. Registered on 27 May 2010.

## Background

Commonly in a surgical randomised controlled trial (RCT) the experimental treatment is a relatively new technique which the surgeons – both individually and as a community – may still be learning, while the control treatment is a well-established standard. One of the primary concerns when designing and analysing a surgical RCT is that this disparity between the levels of expertise that the participating surgeons have in each treatment may distort the comparison between the treatments, potentially leading to biased treatment-effect estimates [1–4].

The need to account for learning effects in surgical RCTs has long been recognised [1–4], and approaches for minimising and accounting for the potentially confounding effects of learning have been developed in the methodological literature [3]. However, there has been limited uptake of these methods and few examples of their application in surgical trial literature [5]. In this paper we present the details of implementing these approaches in a practical example of a surgery RCT to complement the more methodological papers such as Ramsay and Cook’s [3]. Specifically, we demonstrate that the results would have been vulnerable to the confounding effects of learning if these methods were not implemented, validating the concerns that motivated the development of the methodology. We demonstrate the value that these methods bring, and discuss points of consideration for their use in future surgical trials.

The practical example is the Robotic vs. Laparoscopic Resection for Rectal Cancer (ROLARR) trial, funded by the National Institute of Health Research (NIHR) Efficacy and Mechanism Evaluation Programme (EME) programme. The aim of the trial was to compare the safety, efficacy and short- and long-term outcomes of robotic-assisted as compared to standard laparoscopic rectal cancer surgery. In contrast to standard laparoscopic surgery, robotic-assisted surgery was not a well-established approach at the beginning of the trial, with many participating centres obtaining their first surgical robot close to the time at which they began participating in the trial. This disparity only accentuated the need to implement measures of estimating and adjusting for the learning effects.

## Methods

### The ROLARR trial

ROLARR was an international, multicentre, randomised, unblinded, parallel-group trial comparing robotic vs. laparoscopic surgery for the curative treatment of rectal cancer. The trial received national ethical approval in the United Kingdom or either local Ethical Committee/Institutional Review Board approval at international centres. An independent Trial Steering Committee and Data Monitoring and Ethics Committee oversaw the trial conduct. All participants provided written, informed consent. The trial design has been reported previously [6].

Consenting patients were randomised to receive either laparoscopic mesorectal resection as per standard practice (referred to as ‘laparoscopic surgery’ hereafter) or robotic-assisted laparoscopic mesorectal resection (referred to as ‘robotic surgery’ hereafter), which included both totally robotic operations and hybrid operations involving the use of standard laparoscopic techniques for some aspects of the otherwise robotic-assisted operation.

Randomisation (minimisation incorporating a random element) was on a 1:1 basis and was stratified by treating surgeon and other selected prognostic patient factors such as sex and Body Mass Index (BMI). This meant that each participating surgeon in ROLARR was required to perform both robotic and laparoscopic surgery on their trial patients.

The primary aim of the trial was to compare robotic surgery to laparoscopic surgery for rectal cancer resection in terms of the technical difficulty of the operation. The primary endpoint was intra-operative conversion to open surgery, which is a binary indicator of technical difficulty of the operation, and thus lower odds of conversion to open would imply a less technically challenging operation. A reduction of 50% to the odds of conversion to open was considered to be the minimum clinically important difference.

### Design considerations for potential learning effects

In order to minimise confounding due to learning effects, a surgeon inclusion criterion mandating a minimum level of experience in each technique was included. The aim of this was to ensure that all participating surgeons were experts at both techniques, in the sense that they would not still be in the process of learning either of the techniques while contributing to the trial, ultimately leading to a fair comparison between two expertly performed techniques at analysis. At the design stage, the nature of the learning curve in robotic surgery had relatively little evidence from which to derive our inclusion criteria; the evidence base for laparoscopic learning curve was much stronger [7]. Furthermore, the feasibility of recruitment to the trial had to be considered. As the mandated minimum level of experience required increases, recruitment to the trial becomes restricted to a smaller pool of surgeons. Therefore, a pragmatic balance between minimising the chances of learning effects via greater mandated minimum surgeon experience and not compromising feasibility of recruitment had to be found. Ultimately, participating surgeons had to perform a minimum of 30 minimally invasive (laparoscopic or robotic) rectal cancer resections before taking part in the trial, of which at least 10 had to be laparoscopic and at least 10 robotic resections.

In addition to this, data were collected periodically throughout the trial to capture the level of experience of every participating surgeon in performing each of the interventions. All participating surgeons were asked to report the number of previous laparoscopic operations and the number of previous robotic operations that they had performed prior to randomising their first patient. Furthermore, throughout the recruitment period participating surgeons were asked at approximately 3-monthly intervals to report how many laparoscopic and how many robotic operations they had performed – including operations performed outside of the ROLARR trial – since their last reported figure. For each patient, the level of experience of their operating surgeon at the time of their operation was calculated assuming that the operation times within each interval between reported figures were uniform. For example, for a patient whose operating surgeon had reported 98 laparoscopic operations and 56 robotic operations performed up to 30 June 2014, and a further three laparoscopic and one robotic operations between 1 July 2014 and 30 September 2014, and whose operation was on 24 September 2014 – 86 days into the 92-day interval between data captures for their surgeon – the derived number of laparoscopic operations performed by the operating surgeon at the time of the operation was 98 + 3×(86/92) = 100.8, and similarly for robotic, 56 + 1×(86/92) = 56.9.

### Statistical analysis

The primary analysis included the use of multi-level logistic regression to estimate the odds ratios for conversion to open surgery between the treatment groups adjusting for the stratification factors as fixed effects, except for operating surgeon which was adjusted for as a random effect via a random intercept term.

The primary analysis did not include an adjustment for the derived experience levels in laparoscopic and robotic surgery of the operating surgeon at the time of operation for each patient. The analysis of learning effects extended the primary analysis model to include both number of previous laparoscopic operations performed and number of previous robotic operations performed by the operating surgeon as main effects and also as interactions with the treatment effect. Both a main effect and an interaction effect with treatment for each of robotic and laparoscopic learning was forced into the model so that the effects of learning could be estimated in each treatment arm separately. The main effects estimate how learning affects outcomes in the laparoscopic arm, while the interaction effects allow us to estimate how learning affects outcomes in the robotic arm. To parsimoniously account for the fact that the effect of learning may be non-linear, fractional polynomials of degree 1 were used to explore non-linear robotic and laparoscopic effects by including power parameters for each and selecting values for these parameters from a pre-specified restricted space – {−2, −1, −0.5, 0, 0.5, 1, 2, 3} – which maximised the likelihood (minimised the deviance) of the model, as outlined by Royston and Altman [8] and Royston and Sauerbrei [9]. Note that a power of ‘0’ in this context is defined as the natural log function. The learning effects variables were also scaled and centred at their mean values to improve the interpretability of the corresponding regression coefficient estimates. The Stata (v13) command fp was used, and the selected fractional polynomial model was fitted in SAS v9.4, for consistency with the primary analysis.

Both the primary analysis and the learning effects analysis were complete case analyses.

Missing data were minimal, and so sensitivity analyses to quantify the potential impact of missing data on the learning effects model were performed via brute-force model re-fitting under a large number of potential values of the missing fields.

Overly influential observations were identified for each of the regression coefficients for treatment, laparoscopic experience, robotic experience and the interaction terms between treatment and laparoscopic and robotic experience in the learning effects analysis model via the derivation of exponentiated delta-betas. The exponentiated delta-beta is calculated for each regression coefficient for each patient, e.g. for patient i, the exponentiated delta-beta for the treatment-effect regression coefficient is:

$$ \exp \left({\beta}_1^{(i)}-{\beta}_1\right)=\frac{\exp \left({\beta}_1^{(i)}\right)}{\exp \left({\beta}_1\right)}, $$

where *β*_1_ is the regression coefficient for the treatment effect in the full model and ${\beta}_1^{(i)}$ is the treatment-effect regression coefficient in the model where patient i has been omitted. Note that this is the ratio of the estimated odds ratios from the two models, e.g. an exponentiated delta-beta for the treatment effect for patient i of 1.05 would imply that the omission of patient i increases the treatment-effect odds ratio estimate by 5% compared to the inclusion of patient i.

All analyses were pre-specified and were conducted on the intention-to-treat (ITT) population, i.e. all randomised patients were accounted for in the analyses, and patients were categorised into treatment groups based on their randomisation regardless of what they subsequently received. Estimates and their corresponding 95% confidence intervals (CI) are presented. Analyses were carried out in SAS v9.4 and Stata 13.

## Results

**Fig. 1:**
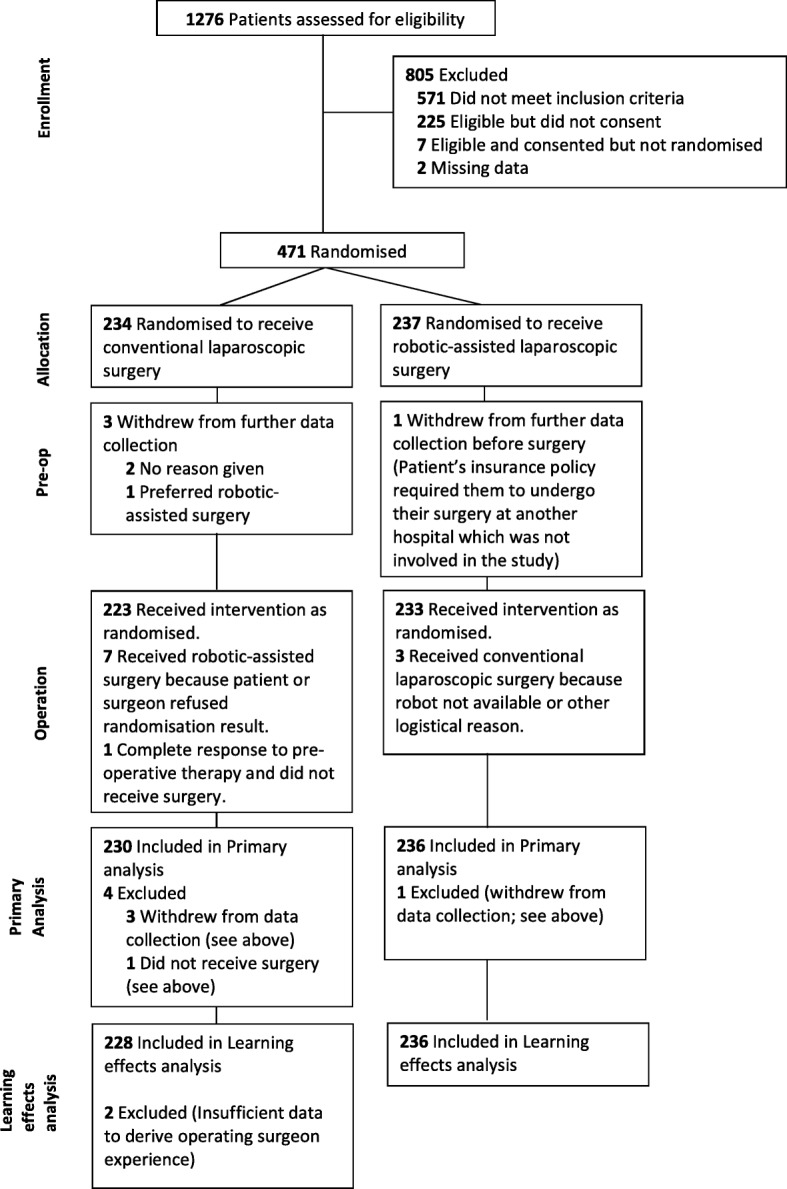
Diagram showing the flow of participants

Figure 1 presents details on participant flow. A total of 471 patients were randomised, of which 466 (98.9%) were included in the primary analysis; reasons for withdrawals are given in Fig. 1. Four hundred and sixty-four of the 466 patients from the primary analysis were included in the learning effects analysis; two patients were excluded due to missing experience data for their operating surgeons. The two treatment groups were well balanced in terms of patient baseline characteristics (Table 1).

**Table 1:** Patient baseline characteristics and crude outcome data

	Standard laparoscopic surgery (*n* = 234)	Robotic- assisted laparoscopic surgery (*n* = 237)	Total (*n* = 471)
Gender			
Male	159 (67.9%)	161 (67.9%)	320 (67.9%)
Female	75 (32.1%)	76 (32.1%)	151 (32.1%)
BMI classification			
Underweight/normal	87 (37.2%)	93 (39.2%)	180 (38.2%)
Overweight	92 (39.3%)	90 (38.0%)	182 (38.6%)
Obese	55 (23.5%)	54 (22.8%)	109 (23.2%)
Neo-adjuvant therapy			
Yes	103 (44.0%)	109 (46.0%)	212 (45.0%)
No	131 (56.0%)	128 (54.0%)	259 (55.0%)
Intended procedure			
High anterior resection	34 (14.5%)	35 (14.8%)	69 (14.6%)
Low anterior resection	158 (67.5%)	159 (67.1%)	317 (67.3%)
Abdominoperineal resection	42 (17.9%)	43 (18.1%)	85 (18.0%)
Conversion to open surgery (outcome)			
Yes	28 (12.2%)	19 (8.1%)	47 (10.1%)
No	202 (87.8%)	217 (91.9%)	419 (89.9%)
Missing	4	1	5

Percentages given are calculated using non-missing data only. Note that out of the factors presented in this table, only ‘conversion to open surgery (outcome)’ had missing data. *BMI* Body Mass Index

**Fig. 2:**
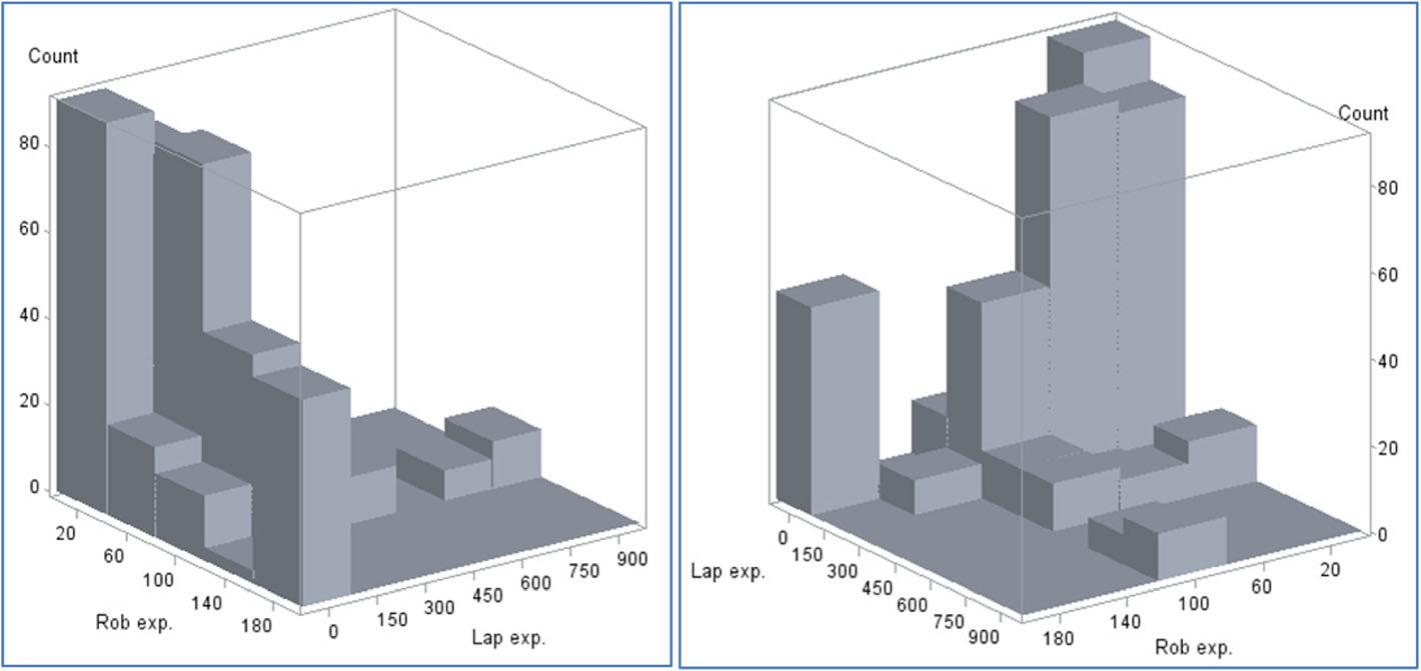
Histogram of the bivariate distribution of number of laparoscopic and robotic procedures performed before the current operation (viewed from two different angles)

The number of previous robotic and number of previous laparoscopic operations performed by the operating surgeon at the time of operations was evaluated for each patient (see ‘Methods’ section). The marginal summaries of these measures over patients are presented in Table 2 and the bivariate distribution of these measured over patients is visualised in a histogram in Fig. 2. The average (median) patient in ROLARR received an operation from a surgeon with experience of 91.4 (interquartile range (IQR) 44.9, 180.1) previous laparoscopic and 49.5 (IQR 30.4, 101.3) previous robotic rectal cancer operations. As seen in Fig. 2, the large majority of operations were carried out by an operating surgeon with experience lying within the region bounded by 10–100 previous robotic and 10–180 previous laparoscopic operations, with several clusters of observations lying on the peripheries at high-robotic-low-laparoscopic and high-laparoscopic regions. Conversions to open surgery were infrequent; 47/466 (10.1%) operations were converted to open, 28/230 (12.2%) in the laparoscopic group and 19/236 (8.1%) in the robotic group (Table 1). This made conversions to open surgery for patients whose operating surgeon’s experience level was in these more peripheral regions capable of producing overly influential observations in the learning effects analysis model.

**Table 2:** Number of laparoscopic and robotic procedures performed before the current operation, summarised across patients

	Number of. previous laparoscopic procedures performed by operating surgeon	Number of previous robotic procedures performed by operating surgeon
	(*n* = 464)	(*n* = 464)
Mean (SD)	152.5 (178.38)	67.9 (48.75)
Median (range)	91.4 (10.0, 853.0)	49.5 (10.3, 183.0)
Interquartile range	(44.9, 180.1)	(30.4, 101.3)

### Primary analysis (not adjusting for learning effects)

The primary analysis model yielded an adjusted odds ratio of 0.614 (95% CI 0.311, 1.211; *p* = 0.16), suggesting that the odds of conversion to open is reduced by around 38.6% (95% CI -21.1%, 68.9%) under robotic surgery compared to laparoscopic. However, there is insufficient evidence to conclude that robotic surgery reduces the odds of conversion, since the confidence interval includes 1.

### Learning effects analysis

Fractional polynomial fitting determined that the functional forms for the robotic and laparoscopic learning effects in the model which best fitted the data were natural log and cubed, respectively. The regression coefficient estimates from the model resulting from adding log robotic and cubic laparoscopic learning effects to the primary analysis model (referred to hereafter as the learning effects model) is presented in Table 3, and the corresponding odds ratios are presented in Table 4.

**Table 3:** Estimated regression coefficients (log-odds ratios), 95% confidence intervals and Wald test *p* values for the treatment and learning effects from the primary analysis and learning effects models

Model	Effect	Adjusted estimate	95% confidence interval for adjusted estimate		*p*
			Lower	Upper	
Primary analysis model	Robotic surgery (vs. laparoscopic)	− 0.488	− 1.168	0.191	0.158
Learning effects model	Robotic surgery (vs. laparoscopic)	− 0.916	− 1.784	− 0.049	0.039
	Surgeon’s robotic experience (log; 1-unit increase)	0.074	− 0.706	0.854	0.852
	Surgeon’s laparoscopic experience (cubic; 10^8-unit increase)	0.309	− 1.1033	0.4852	0.445
	Interaction: surgeon’s robotic experience (log; 1-unit increase) × robotic surgery	− 1.076	− 2.110	− 0.041	0.042
	Interaction: surgeon’s laparoscopic experience (cubic; 10^8-unit increase) × robotic surgery	− 0.160	− 2.153	1.833	0.875

**Table 4:** Estimated adjusted odds ratios (robotic vs. laparoscopic) for conversion to open surgery vs. operating surgeon’s level of previous laparoscopic and robotic experience

Effect	Surgeon’s laparoscopic experience level (no. of previous operations)	Surgeon’s robotic experience level (no. of previous operations)	Adjusted odds ratio (robotic vs. laparoscopic)	95% confidence interval for adjusted odds ratio	
				Lower limit	Upper limit
Primary analysis model Robotic surgery (vs. laparoscopic)	–	–	0.614	0.311	1.211
Learning effects model Robotic surgery (vs. laparoscopic)	45	30	0.969	0.431	2.178
		50	0.559	0.264	1.185
		100	0.265	0.084	0.840
	91	30	0.968	0.432	2.172
		50	0.559	0.265	1.180
		100	0.265	0.084	0.836
	180	30	0.960	0.431	2.138
		50	0.554	0.267	1.151
		100	0.263	0.085	0.814

The learning effects model yields an estimated odds ratio for a patient whose operation is being performed by a surgeon with the mean experience level in ROLARR – 152.46 previous laparoscopic and 67.93 previous robotic operations performed – of 0.40 (95% CI 0.168, 0.953; *p* = 0.039).

Increasing operating surgeon laparoscopic and robotic experience are estimated to have no notable effect on the odds of conversion under laparoscopic surgery. This is clear from the main effects estimates presented in Table 3, which have negligible magnitude and non-significant Wald tests. The effect of increasing operating surgeon laparoscopic experience is also estimated to have no notable effect on the odds of conversion under robotic surgery. This is reflected by the small effect size and non-significant Wald test for the interaction term for laparoscopic experience and treatment in Table 3. However, the effect of increasing operating surgeon robotic experience is clearly different under robotic surgery compared to laparoscopic surgery. This is clear from the significant Wald test for the interaction term for robotic experience and treatment in Table 3. The model suggests that the treatment-effect odds ratio (robotic/laparoscopic) decreases by a factor of 0.341 (95% CI 0.121, 0.960; *p* = 0.042) per unit increase in log-number of previous robotic operations performed by the operating surgeon.

**Fig. 3:**
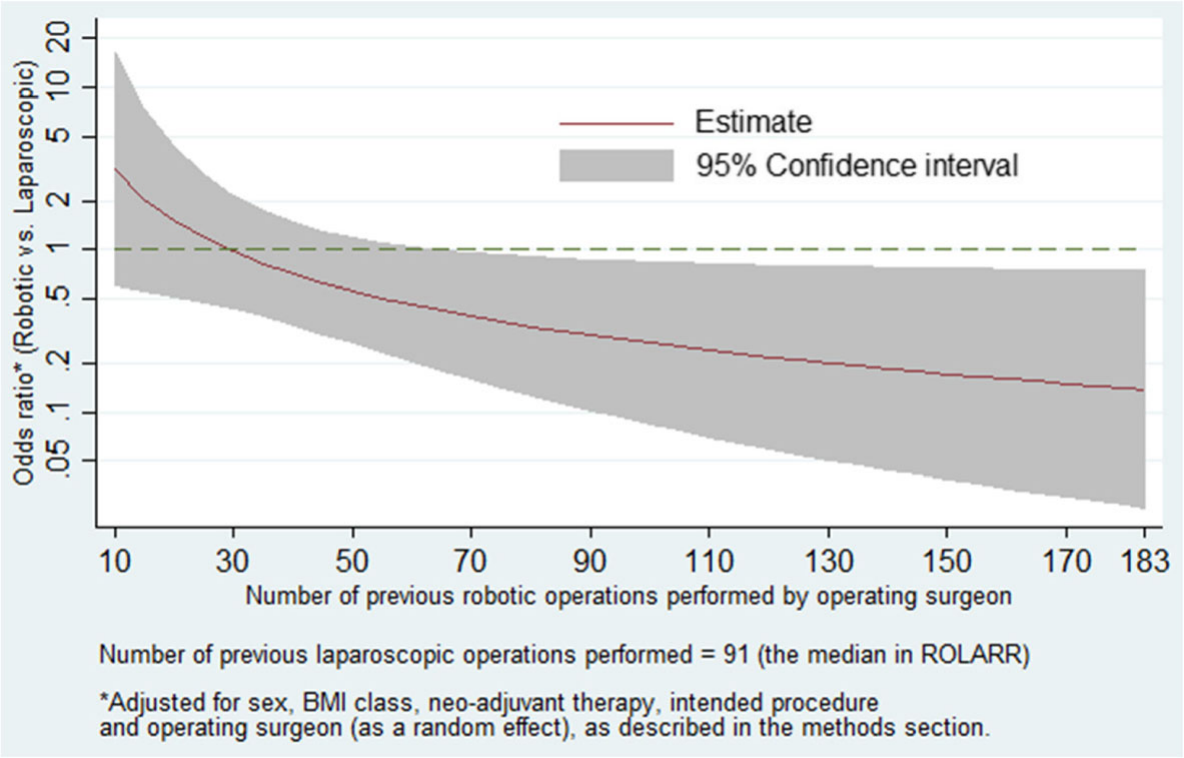
Graph of estimated odds ratio (robotic vs. laparoscopic) and 95% confidence interval for conversion to open surgery vs. operating surgeon previous robotic experience at the median level of laparoscopic experience

Table 4 presents the estimated treatment-effect odds ratio at various levels of operating surgeon laparoscopic and robotic experience, and illustrates that increasing operating surgeon robotic experience notably affects the treatment-effect odds ratio in favour of robotic surgery regardless of the level of laparoscopic experience. Figure 3 presents the estimated treatment-effect odds ratios across the entire range of robotic experience levels observed in ROLARR while fixing laparoscopic experience at 91 (the median). It shows a clear reduction in the odds ratio as robotic experience increases. In particular, at levels of robotic experience beyond 70 previous operations, the model estimates clinically meaningful effect sizes (odds ratios less than 0.5) which are statistically significant (confidence intervals not spanning 1).

**Fig. 4:**
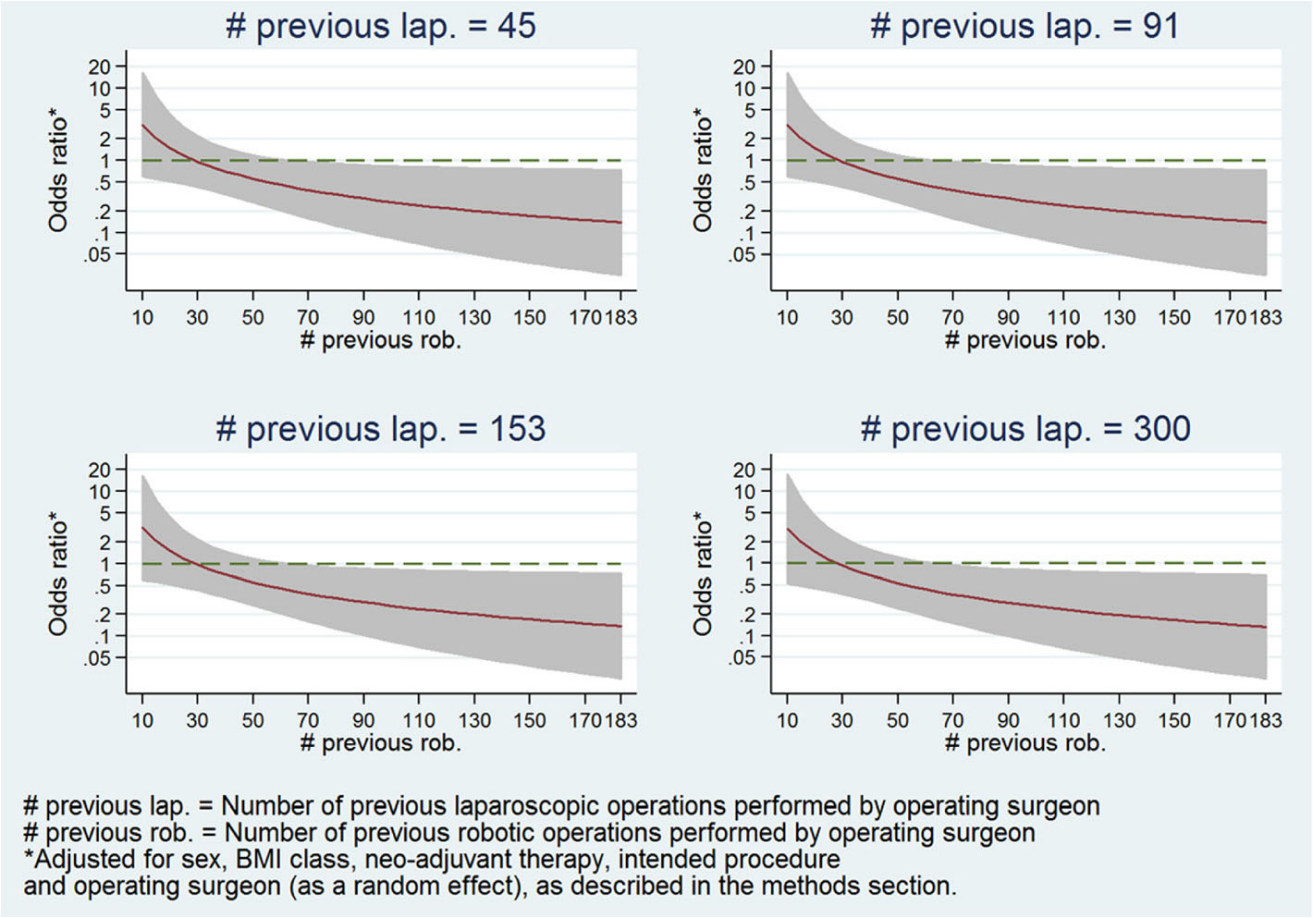
Panel plot of estimated odds ratio (robotic vs. laparoscopic) and 95% confidence interval for conversion to open surgery vs. operating surgeon previous robotic experience at various levels of laparoscopic experience

**Fig. 5:**
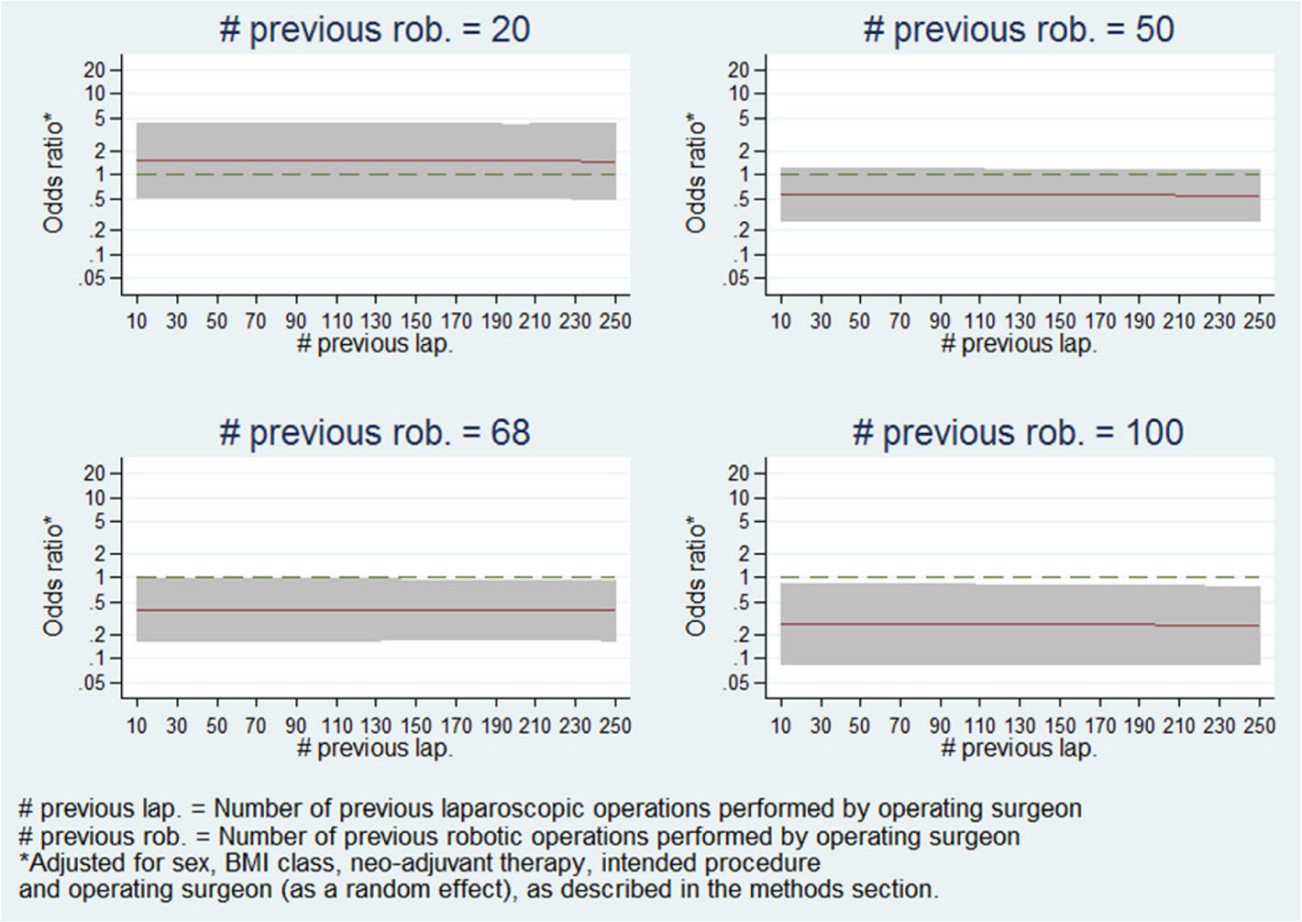
Panel plot of estimated odds ratio (robotic vs. laparoscopic) and 95% confidence interval for conversion to open surgery vs. operating surgeon previous laparoscopic experience at various levels of robotic experience

Furthermore, Fig. 4 presents the estimated treatment-effect odds ratios across the entire range of robotic experience levels at various selected levels of laparoscopic experience, and demonstrates that the relationship seen in Fig. 3 holds regardless of laparoscopic experience level. Figure 5 presents estimates of treatment-effects odds ratios across a range of laparoscopic experience levels at various selected levels of robotic experience, and demonstrates that the estimated effect of laparoscopic experience on the treatment effect is negligible.

### Learning effects analysis: missing data

In order to test the robustness of the model against potential effects of excluding the two patients with missing data, sets of values of laparoscopic and robotic operating surgeon experience were assumed for the two patients and the model repeatedly re-fitted under various values. In total this was done 625 times to cover all possible combinations of number of previous laparoscopic operations in {10, 45, 91, 180, 500} and number of previous robotic operations in {10, 30, 50, 101, 183} for each patient. None of the re-fitted models yielded estimates that were notably different to the model which excluded these two patients. The distributions of estimates from these 625 models are summarised in Table 5.

**Table 5:** Summary of regression coefficients and standard errors produced by the 625 models which incorporated a range of imputed operating experience data for the two patients who had missing data (see ‘Methods’ section)

Parameter	Original learning effects model parameter estimate	Median and range of parameter estimates from the 625 models of imputed data
Treatment effect: estimate	− 0.916	− 0.946 (− 0.964, − 0.908)
Treatment effect: standard error	0.441	0.441 (0.438, 0.443)
Laparoscopic experience: estimate	− 0.309	− 0.317 (− 0.338, − 0.205)
Laparoscopic experience: standard error	0.404	0.408 (0.349, 0.409)
Robotic experience: estimate	0.074	0.073 (− 0.080, 0.168)
Robotic experience: standard error	0.397	0.392 (0.382, 0.397)
Treatment × laparoscopic experience interaction: estimate	− 0.160	− 0.144 (− 0.226, − 0.135)
Treatment × laparoscopic experience interaction: standard error	1.014	1.012 (0.989, 1.015)
Treatment × robotic experience interaction: estimate	− 1.076	− 1.077 (− 1.149, − 0.970)
Treatment × robotic experience interaction: standard error	0.526	0.524 (0.518, 0.527)

### Learning effects analysis: highly influential observations

To explore whether the learning effects analysis model results were being disproportionately determined by overly influential observations from regions of sparse data, the exponentiated delta-betas for the treatment effect, main effects of laparoscopic and robotic experience, and interactions between treatment effect and laparoscopic and robotic experience were calculated. Several overly influential observations were identified. For example, the most influential observation on the robotic experience by treatment-effect interaction term had an exponentiated delta-beta of 0.730. When the model was fitted omitting that patient, it estimated that the treatment-effects odds ratio (robotic/laparoscopic) decreases by a factor of 0.249 (95% CI 0.080, 0.773; *p* = 0.0163) per unit increase in log-number of previous robotic operations performed by the operating surgeon, rather than the factor of 0.341 (95% CI 0.121, 0.960; *p* = 0.042) reported above. That patient was operated on by a surgeon with 169.6 previous robotic and 21.02 previous laparoscopic operations – in a peripheral area of experience with sparse data (see Fig. 2) – and was the only conversion to open surgery out of the 49 patients operated on by two surgeons who lie in that region. All of the overly influential observations were similar to this example: legitimate observations, conversions to open and with operating surgeon experience towards the extremities of the observed distribution, in areas of relatively sparse data.

**Fig. 6:**
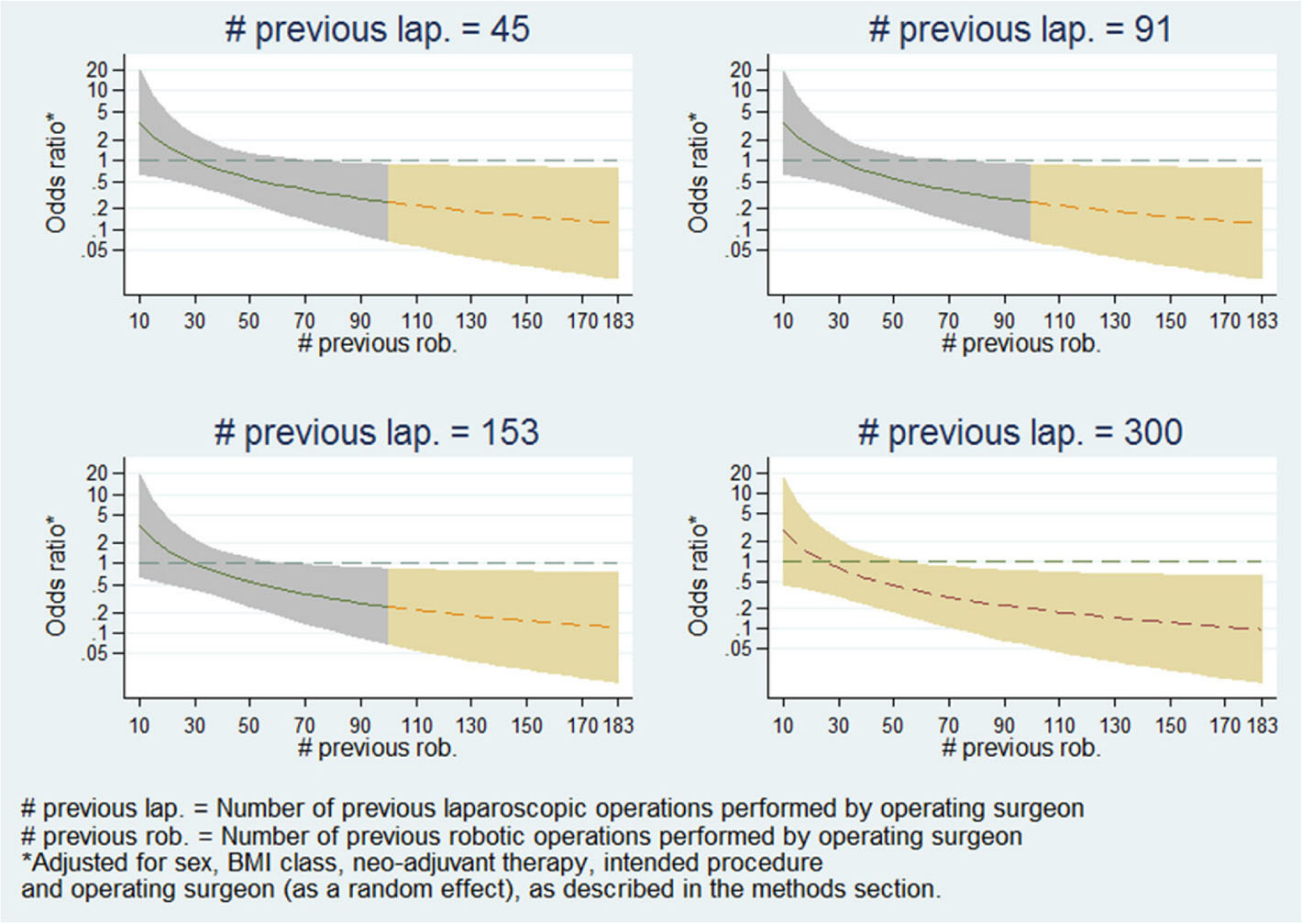
Panel plot of estimated odds ratio (robotic vs. laparoscopic) and 95% confidence interval for conversion to open surgery vs. operating surgeon previous robotic experience at various levels of laparoscopic experience (fitted on subsample of patients). This model was only fitted on patients whose operating surgeon had <= 101.3 previous robotic operations and <=180.1 previous laparoscopic operations. The graphs have been split by colour to show the model estimates where the actual data is and the model estimates which are extrapolations

**Fig. 7:**
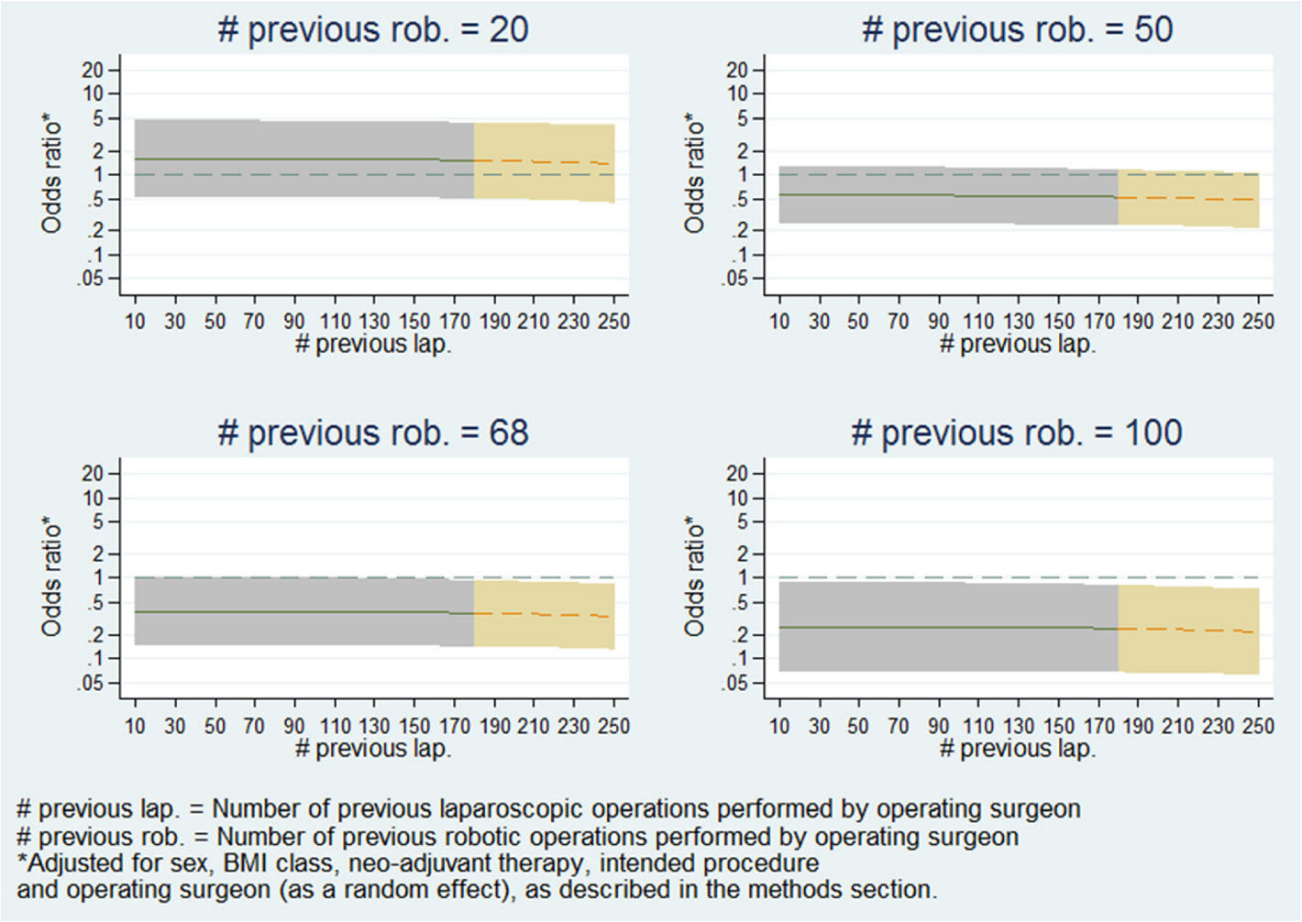
Panel plot of estimated odds ratio (robotic vs. laparoscopic) and 95% confidence interval for conversion to open surgery vs. operating surgeon previous laparoscopic experience at various levels of robotic experience (fitted on subsample of patients). This model was only fitted on patients whose operating surgeon had <= 101.3 previous robotic operations and <=180.1 previous laparoscopic operations. The graphs have been split by colour to show the model estimates where the actual data is and the model estimates which are extrapolations

A concern which naturally develops from that is that if the model estimates are being highly influenced by data in the extremities of the distribution of experience levels, then the model may not be an accurate representation of the majority of ROLARR participants. Thus, in order to explore the robustness of model estimates, the model was fitted again including only patients whose operation was performed by a surgeon whose laparoscopic and robotic experience levels were lower than the upper quartiles – i.e. between 10 and 101.3 previous robotic and between 10 and 180.1 previous laparoscopic operations. This limited model yielded very similar estimates. The estimated relationships of robotic experience vs. treatment effect and laparoscopic experience vs. treatment effect from this re-fitted model are presented in Figs. 6 and 7, and are clearly very similar to the relationships seen from the full model in Figs. 4 and 5.

## Discussion

The primary analysis for ROLARR provided insufficient evidence of superiority of robotic surgery. The estimated odds ratio did not show a clinically important reduction in the odds of conversion to open under robotic surgery compared to laparoscopic. In isolation, the primary analysis suggests that robotic surgery, as compared with laparoscopic surgery, does not significantly reduce the risk of conversion to open surgery when performed by surgeons with varying experience of robotic surgery.

The results of the learning effects analysis suggest that participating surgeons in ROLARR were already experts in laparoscopic surgery with respect to the need to convert to open surgery. This is clear from the negligible estimated effect of increasing operating surgeon laparoscopic surgery experience on both the conversion rate in the laparoscopic arm and on the difference between arms, adjusting for prognostic patient factors as well as other operating surgeon factors (caught by adjusting for operating surgeon as a random effect). However, it seems that the surgeon inclusion criterion did not fully accomplish the aim of ensuring that all participating surgeons were experts in both treatments, since the results also suggest that some, if not all, participating surgeons in ROLARR were still learning robotic surgery. This is clear from the notable effect of increasing operating surgeon robotic surgery experience on both the conversion rate in the robotic arm and on the difference between arms, again adjusting for prognostic patient factors as well as other operating surgeon factors (caught by adjusting for operating surgeon as a random effect).

Furthermore, the nature of the relationship between operating surgeon robotic experience and the treatment effect suggest that the desired clinically important difference between the treatments occurs at higher levels of robotic surgery experience than the average (median) in ROLARR. Given this, it could be argued that the primary analysis represents a comparison of laparoscopic surgery and robotic surgery when performed by a surgeon who is an expert at laparoscopic surgery, but still learning robotic surgery. While this may be representative of clinical practice during the period over which ROLARR took place, it could be argued that it does not tell us the whole story for the purpose of policy-making.

The learning effects analysis paints a clear picture, suggesting that robotic surgery does in fact confer an advantage compared to laparoscopic surgery in terms of the risk of converting to open surgery when the operating surgeon has more substantial previous experience with robotic surgery. Model diagnostics and sensitivity analyses for the learning effects model have shown it to be a significantly better fit than the primary analysis model, and have also shown it to be robust to the potential effects of the two excluded patients and to the effects of highly influential observations.

### Limitations

One limitation of the learning effects analysis in ROLARR is that the experience variables were fitted as fixed effects in the model. This imposes the implicit assumption that every surgeon has exactly the same learning curve. This may be an overly strong assumption and it may be more appropriate to include random effects for the experience variables – both their main effects and their interactions – which in particular would allow for the possibility that different surgeons learn at different rates and plateau at different levels of proficiency. In this particular case, attempting the inclusion of random effects led to model convergence issues.

Another limitation is that the presented learning effects model only accounts for learning on an individual level, derived only from the number of previous operations performed, and assumes that proficiency is monotonic non-decreasing. The former is a simplification of a complex mechanism by which an individual surgeon’s proficiency is affected via multiple sources including learning at an expert community level as well as from sheer case volume [4, 10, 11], e.g. a surgeon with 30 operations spread out over 3 years is perhaps not going to be as proficient as a surgeon with 30 cases performed over 3 months, all else being equal. The latter is a simplification of the nature of learning, which can involve deteriorations in proficiency; e.g. due to length of time since the most recent operation [10]; e.g. a surgeon with 100 previous cases, but who has not performed a case in over 5 years, may be expected to be less proficient than a surgeon with 100 previous cases all performed within the last year, all else being equal.

## Conclusions

The learning effects analysis presented suggests, in contrast to the primary analysis, that robotic-assisted laparoscopic surgery does confer an advantage over standard laparoscopic surgery in terms of the risk of conversion to open surgery, when performed by an operating surgeon with a substantial level of previous experience with robotic surgery, regardless of their level of previous experience in standard laparoscopic surgery.

Beyond the ROLARR trial, in this paper we have demonstrated the implementation of these approaches for accounting for learning in a practical example of a surgery RCT analysis which would otherwise have been vulnerable to the confounding effects of learning. The results demonstrate the value of implementing such approaches since we can see that without them the analysis would indeed have been confounded.

## Abbreviations

BMI: Body Mass Index
CI: Confidence interval
EME: Efficacy and Mechanism Evaluation Programme
IQR: Inter-quartile range
ITT: Intention-to-treat
NIHR: National Institute of Health Research
RCT: Randomised controlled trial
ROLARR: Robotic vs. Laparoscopic Resection for Rectal Cancer
